# Cases in the Practice of an Hospital Physician

**Published:** 1814-07

**Authors:** 


*For the Medical and Physical Journal.*

### Cases *in the* Practice *of an* Hospital Physician.

### *Case of Ascites.*

D. E. aged 13, admitted the 23d of March. About six weeks ago, after complaining much for some time of a pain in her stomach, thirst, and difficulty in passing her urine, which she likewise passed in smaller quantity than usual, began to perceive a swelling in her belly, which, since that time, has been gradually increasing, and is now pretty considerable. The swelling is diffused equally over all her belly, and there is an evident fluctuation in it; her thirst and scarcity of urine still continue, though not to such a degree as at first; P. 112; appetite bad; belly regular; has no symptoms which indicate the approach of her menses; has used a variety of remedies, but no diuretics.

24th.— ℞. Aq. Cinnam. font. a. ℥ſs.

Oxym. Colchici. ʒi. M. Capt. ter de die vacuo ventriculo.

℞. Cr. Tart. ℥ſs.

Syr. Simp. q. s. f. Elect.

Sum. Singul. dieb. q. v.

Let her belly be rubbed three times a-day with Ol. Camph. and covered with flannel.

25th.

25th.—P. 82. As formerly. Let her measure the size of her belly from time to time.

26th.—P. 94, and very small. Thinks the quantity of her urine increased. Contin.

27th.—P. 84. The swelling of the upper part of her belly rather diminished. Had six stools yesterday. Contin. Let her have some tamarinds to take occasionally. Let her have an egg for supper, and a bit of meat for dinner.

28th.—The swelling continues to diminish, and the urine to increase. Contin.

29th.—P. 84.—The swelling much as it was, but the quantity of her urine continues to increase. Contin.

30th.—The swelling of her belly continues to increase, and the quantity of her urine to increase. Contin.

April 4th.—No change in her swelling for some days, but has not got her medicines regularly.

5th.—Swelling rather less; her medicines generally give her five or six stools daily.

10th.—The swelling continues to fall, but she complains to-day of head-ach, sickness, and diminished appetite. Sum. Pulv. Ipecac. gr. viij. vesp. Int. alia Med. h. n.

11th.—Vomit operated well. Head-ach and sickness abated. Rep. Med. ut antea.

12th.—P. 80. As before. Cont. Med.

14th.—Swelling continues to abate, and urine increasing. Contin.

15th.—Symptoms continue to mend. Om. Colch. perstet in usu Chrystal. Tart.

16th.—As yesterday. Contin. Elect.

17th.—The swelling seems to be increased, and her urine somewhat diminished; her stools continue as before. Rep. Colch. Contin. Elect.

18th.—Swelling again a little diminished, and urine a little increased. Contin. Med.

24th.—Has still some hardness in the lower part of her belly. Contin. Med.

28th.—Dismissed cured. Let her have some of the Oxym. Colch. and Electuary with her, of which it is advised her to continue in the use for some time.

### *Case of Pyrosis.*

A. I. aged 25, admitted the 23d of March, for two years past has been affected more or less with a swelling in the region of her stomach, which sometimes rises to a considerable height, causing great pain and sickness, and depriving her altogether of her appetite. This swelling used formerly to subside at times, but for more than two months past has never subsided; it gives her great pain when pressed, and sometimes, though rarely, she belches up wind with relief. She throws up clear water, which is very sour, and, as she says, feels as hot as boiling water for the most part; is costive; has been married two years, and bore a child above a year ago. Her menses appeared lately in small quantity, but have, for some time pas , been irregular. Pulse natural.

24th.—℞. Al. Socot. Extract. Gent. a. ℈i.

Sal. Mart. ʒſs.

Syr. Simp. q. s. fiant. Pil. xxx. quarum sum. ij, om. noct

25th.—Complains much of pain, sickness of her stomach, and difficulty of breathing.

℞. Aq. Menth. Pip. font. a ℥iij.

Magnes. alb. ℈ij.

Pulv. Zing. ʒſs. M.

Sum. ℥iſs. bis de die.

Contin. Pil. Applic. Emp. de Cicut. Reg. Ventris.

26th.—Pains of her stomach easier.

℞. Extract. Cicut. ℈ij. form. in Pil. xl.
Sum. i. m. and v.
Contin. alia Medicamenta.

27th.—Swelling of her stomach much diminished. Contin. Let her have the same diet ordered for D. E.

28th.—Complains of sickness and pain of her side. Let it be rubbed with Linim. Vol. and covered with flannel.

29th.—Pain of her side relieved. Contin.

30th.—Swelling of her stomach much less, and every way easier. Cont.

April 2d.—Let her have fresh plaister for her stomach.

3d.—Dismissed without complaint. Let her have a plaister larger than the former, and a box of her laxative pills.

### *Case of Acute Rheumatism.*

M. N. aged 17, admitted the 29th of March, on the 24th instant was seized with coldness and trembling, succeeded by heat and thirst, head-ach, vertigo, and other febrile symptoms; has complained all along of severe pains in her back, in the top of her shoulders, and in her knees; belly bound; has never menstruated; P. 120. This patient was admitted into the hospital about the beginning of the year with the same complaints, which were treated with repeated bleeding, sweating, &c. and after a fortnight was dismissed cured.

Injiciatur statim enema domesticum. & Sum. Solut. Tart. Emet. ℥ſs. hor. 7ma vesp. more solito at ten o'clock at night.

P. 128. Has vomited freely with two doses of the solution; skin hot and dry, and she complains much of her pains. P. 128. Mittatur Sang. e Brach. ad ℥viij. Statim foveantur Crura.

30th.—P. 128. Was very restless the first part of the night, but slept better towards morning; skin hot; very thirsty; pains still very severe. Mitt. Sang. e Brach. ad ℥vi. st. Rep. Solut. Tart. Emet. vesp. & fov. Crur. more solito. Let her have apple-water to drink, and roasted or boiled apples with panada for food. Inj. Enem. dom. St.

31st.—P. 130. Blood sizy, but the crassamentum very fluid. Threw up some foul viscid stuff with the solution; pains in her limbs easier, but complains much of head-ach, and a pain between her shoulders. Applic. Emp. Lyttæ Nuch. & Sum. vin. rubr. ℔fs. quotid. mixed with her food. Complains of pains in her belly. Inj. En. dom. statim. Contin. Fotus.

April 1st.—P. 134. All her pains easier, except what arise from the irritation of the blistering plaister, of which she complains very much. Let the plaister be removed, and an emollient cataplasm applied to the part. Contin. Fot. & En. dom.

2d.—P. 120. Thirst abated; skin not so hot as it was, and she seems to be easier. Contin. Enem. & Fot.

3d.—P. 120. Took three doses of the Tart. Emet. last night, which vomited her well; skin cool; tongue moist; no thirst; sleeps better. Contin. Enem. & Fot. Sum. Mistur. Salin. ℥ij. 3tia q. q. hor.

4th.—P. 108. Skin cool, and every way better.

5th.—P. 92. Was very delirious in the night, and continued so till ten o'clock this morning, but is now quite sensible. All her pains removed. Tongue clean and moist.

6th.—P. 104. Slept well, and is every way better.

8th.—P. 94. Convalescent.

10th.—P. 100. Has a swelling of the submaxillary glands on the right side. Applic. Emp. Lyttæ pone Aurem dextram.

11th.—The swelling diminished.

12th.—P. 110. Submaxillary glands of the other side swelled. Applic. Emp. Lyttæ ut antea. Costive. Inj. En. dom. St.

13th.—P. 78. The blister has risen well, and the pain is easier.

14th.—P. 92. The swelling of her neck is abated, but the pain still continues, except the frequency. She has hardly any other symptom of fever. Part. Tument. Applic. Catap. ex Mic. Pan. & Lact. addend. pauxil. Ol. Camphor.

15th.—P. natural. The pain of her neck was relieved by the cataplasm. Vesp. Applic. iterum. Cataplasma.

16th.

16th.—Hardly any swelling remains on her neck, and the pain is also gone. Rep. Catapl.

17th.—Neck quite well, but complains of weakness and giddiness. Capt. Infus. Gentianæ ℥ij. man. & hor. ante Prandium.

19th.—Continues to recover. Repetatur Infus. Gent.

20th.—Dismissed cured, with some of the bitters.

### *Case of Trismus.*

A. B. aged 14, admitted the 7th of April. About four years ago, after being violently affected by the death of a near relation, became subject to a variety of nervous or hysterical complaints, such as acute pains of her head and breast, globus hystericus, screamings, fainting fits, or fits of lowness, &c. These at first continued upwards of three months, and have returned at least once every year since that time. Their attacks are said always to have been preceded more or less by symptoms of fever. About four months ago she fell ill in her ordinary way, but at the beginning of this attack, after being considerably feverish for some time, and complaining much of pain and swelling of her throat, which was supposed to have suppurated, she was observed to be affected with the locked jaw, which has continued uniformly till this time, insomuch that her mouth can never be opened without forcing a hard substance between her teeth. About four or five weeks ago, she became delirious for several days, after which she began to be affected with a variety of spasmodic contractions almost of every part of her body, but more remarkably of all the flexor muscles of the upper and lower extremities. These, as well as the locked jaw, still continue very constant and severe. She generally complains of severe pain about the breast or stomach, and, when the spasms are violent, her pains are universal. The faculties of her mind do not seem to be impaired; her pulse is quite natural; she has sometimes a considerable degree of subsultus tendinum; there are as yet no appearances of her menses; has grown very tall of late; takes very little food, and commonly throws up what she does take; is generally costive. Inj. Enem. Dom. vesp. Inung. femor. interna Ung. Merc. ʒij.

8th.—Complains of a little soreness of her teeth. In other respects as yesterday. Let only ʒj. of mercurial ointment, with an equal quantity of Axunge, be rubbed in to-night.

9th.—Took an anodyne last night, but threw it up immediately; pains and contractions rather more severe to-day; costive. Inj. Enem. dom. St. & post horam. Inj. Enem. ex Inf. Lin. ℥iv. Tinct. Opii gtt. l. Contin. Unct.

10th.

10th.—Retained the anodyne injection, and passed an easy night. Rep. Enemata ut heri.

11th.—Continues as yesterday. Contin. Enem. & Unct.

12th.—Passed an easier night than usual; mouth begins to be a little affected. Intermit. Unct. hac noct. Rep. Enem.

13th.—P. 86. As before. Rep. Enem. Purg. & Anod. ut antea.

14th.—P. 84. Symptoms as before; did not retain either of the clysters last night. Vesp. hor. 6tâ Injic. Enem. fœtid. & reddito hoc enemate, post horam unam vel alteram, Inj. Enem. ex Aq. font. ℥ ij. Tinct. Opii, gtt. lx. Continues to spit.

15th.—P. 68. Had a stool by the first clyster last night, but did not retain the second; symptoms continue as before. Omittatur Enema & Capt. Haust. ex Aq. Cinnamon. Syr. Limon. āā ℥ fs. Tinct. Opii, gtt. xxx.

16th.—P. 82. Took very little of the draught last night, and she seemed to retch after it; has had a stool to day. Capt. hor. Som. Tinct. Opii, xxx. ex lacte. Let some electrical sparks be taken from the outside of her wrists.

17th.—Was hardly sensible of the sparks taken from her wrists yesterday; other symptoms as before; she got the Tinct. Opii, but not mixed with milk as prescribed; was more restless than usual last night. Let her have a moderate shock of electricity through each wrist.

18th.—Did not complain of pain from the electrical shocks yesterday, which occasioned a little starting of her whole body, but no motion of the joints to which they were applied. Symptoms continue as before; had a small stool to-day. Let three or four electrical shocks be given to each wrist, and let her have Tinct. Opii gtt. v. every half hour till she has taken it ten or twelve times.

19th.—Would take only three doses of the laudanum. The electricity, though it made her start, did not give her any pain, and had no effect upon the motion of the wrists to which it was applied. Let it be made stronger, and six shocks given to each wrist. Injic. Enem. ex Sal. Com. ℥ j. Reddito hoc enemate. Aq. tepid. ℥ ij. cum Tinct. Opii, gtt. lx.

20th.—Several shocks of electricity, as strong as the machine would admit, were applied, but without seeming to give her any pain, or making any change in the state of the joint; had a stool by the laxative clyster, but is uncertain whether she retained the anodyne; rest much as usual.

℞. Op. gr. ij. divid. in Pil. ij.
Capt. vesp. 2bus vicib.

21st.—Did not reject the pills last night, but they have made no sensible alteration on her symptoms. Vesp. Inj. En. Salis. & h. s. Capt. Opii, gr. iij. in Pil. ij. divis.

22d.

22d.—Had a stool by the clyster yesterday. Kept the pills on the stomach, though she had some retching after taking them; slept better than the night before, but not better than she has usually done; the contractions of her limbs continue the same, and she complains of more than usual sickness this forenoon. P. M. Inj. En. ex Aq. tepid. ℔ij. & h. s. Capt. Op. gr. iij. ut hesternâ nocte.

23d.—Refused to have the clyster yesterday; kept the pills on the stomach at night, but they have had no effect on her symptoms.

℞. Opii, gr. iv. divid. in Pil. ij. hor. s. sumend.

24th.—Slept no better last night than usual; symptoms as before; had no stool yesterday. P. M. Inj. En. Salin. & h. som. Capt. Op. gr. v. in Pil. ij. divis.

25th.—Had a stool by the clyster yesterday; has slept better than usual this morning; continues drowsy to-day; other symptoms much as before. Hor. Som. rep. Opium ad gr. v. ut heri.

26th.—State of symptoms much as yesterday; wrists rather more moveable. Vesp. Inj. Enem. Salin. & h. s. rep. Opium ad gr. iv. in Pil. iij. divis. Had no stool yesterday.

27th.—Had a stool by the clyster, but did not take the pills till this morning. Hor. som. rep. Pil. ut heri prescript.

28th.—Though she took the dose last night as ordered, she has been rather more restless and slept less than usual; symptoms otherwise the same. Capt. statim Opii, gr. iij. in Pil. ij. divis. & h. s. rep. Opium ad gr. vi.

29th.—Became sick yesterday afternoon, and vomited very frequently in the night; still continues sick; other symptoms as before. P. M. Inj. Enem. Salin. omitte Opium.

May 1st.—Had a stool by the clyster yesterday; symptoms much as before. H. s. Capt. Opii, gr. v. in Pil. ij. divis.

2d.—Could not take the pills last night; symptoms as before. P. M. Inj. En. Salin.

3d.—Dismissed at her own desire.

(To be continued.)

